# Droplet Generation During Mastoidectomy and Mitigation by Use of a Novel Barrier Drape

**DOI:** 10.7759/cureus.100442

**Published:** 2025-12-30

**Authors:** Wei Li Neo, Jiunfong Thong, Heng Wai Yuen, Vanessa Tan, Matthew Woo, Hui Shi Ong, Jia Hui Ng

**Affiliations:** 1 Otolaryngology - Head and Neck Surgery, Sengkang General Hospital, Singapore, SGP; 2 Otolaryngology - Head and Neck Surgery, Tseung Kwan O Hospital, Hong Kong, HKG; 3 Otolaryngology - Head and Neck Surgery, Changi General Hospital, Singapore, SGP; 4 Otolaryngology - Head and Neck Surgery, Singapore General Hospital, Singapore, SGP; 5 Clinical Innovation and Technology Unit, Singapore General Hospital, Singapore, SGP

**Keywords:** drape, droplet transmission, mastoid surgery, personal protective equipment, surgical drilling

## Abstract

Objective: This study aims to investigate the degree of contamination generated by mastoid drilling and the extent to which this is mitigated by the use of a novel barrier drape, Otodome, which contains the surgical field.

Study design: Cortical mastoidectomy with a high-speed self-irrigating drill was performed under three test conditions in the cadaveric lab, including A, without a microscope, B, with a microscope alone and C, with a microscope fitted with the Otodome. Schülke Optics fluorescent solution was used as irrigation fluid.

Setting: High-speed irrigation drills used during mastoidectomy create large amounts of droplet dispersion. This study quantifies the amount of droplet dispersion and the extent to which the barrier drape mitigates it.

Methods: The degree (mild, moderate or severe), distribution, and distance of contamination of the surroundings were collected in each test condition. The extent of contamination of the surgeon was noted.

Results: Condition A had the worst contamination of both the surgeon and surroundings, with the furthest distance and heaviest density of droplet spread after a cortical mastoidectomy. Droplets were dispersed up to 290cm from the surgical field. Using the barrier drape in Condition C significantly reduced the droplet dispersion to a maximum of 78cm from the surgical field. There was a significant reduction in the density of contamination of the surroundings. Most of the heavy contamination was confined within the barrier drape. There was no contamination of the surgeon’s face and head region.

Conclusion: Mastoid drilling produces an extensive amount of droplet dispersion. Improvised barrier drapes have been shown to mitigate this problem, but are cumbersome to use. The Otodome is shown to be an effective barrier drape.

## Introduction

Mastoid drilling involves the use of high-speed irrigation drills and creates a large amount of droplets and aerosols, which contaminate not only the surgeon but also the operating theatre and support staff [1,2]. The COVID-19 pandemic has created an increased awareness of the importance of personal protective equipment (PPE). In the post-pandemic era, this increased awareness has created a paradigm shift where it is recognised that droplets generated during mastoidectomies may pose significant risks to the surgical team. Conventional PPE does not adequately protect the surgeon from droplets generated from mastoidectomies. PPE such as safety goggles, face shields, and powered air-purifying respirators affect the surgeon’s use of the surgical microscope to varying degrees [2]. Acknowledging this problem, studies have been published on the recommended personal protective equipment (PPE) for use during mastoid surgery [2]. Lawrence et al. found that the use of a filtering facepiece(FFP3) mask or half face respirator with safety goggles is the most viable option for mastoid surgery [2]. However, the use of ideal PPE often creates problems with visualisation during surgery, especially when used with an operating microscope.

Various studies have proposed the use of barrier drapes around the operative field to reduce the risk of droplet and aerosol dispersion during mastoid surgery [1-7]. As most of the drapes described have been improvised for use from existing drapes made for other purposes [4-7], they may be more tedious to set up and often affect the dexterity of the surgeon. We developed a novel custom barrier drape, the Otodome, devised for use during mastoid drilling. This study aims to illustrate the degree of contamination that is generated by mastoid drilling, and the extent to which the use of the Otodome, devised for the purpose of protecting healthcare workers during mastoid drilling can mitigate this.

## Materials and methods

This experiment was performed at a temporal bone laboratory. This study was exempted from ethics approval by the Singhealth Centralised Institutional Review Board (CIRB).

**Figure 1:**
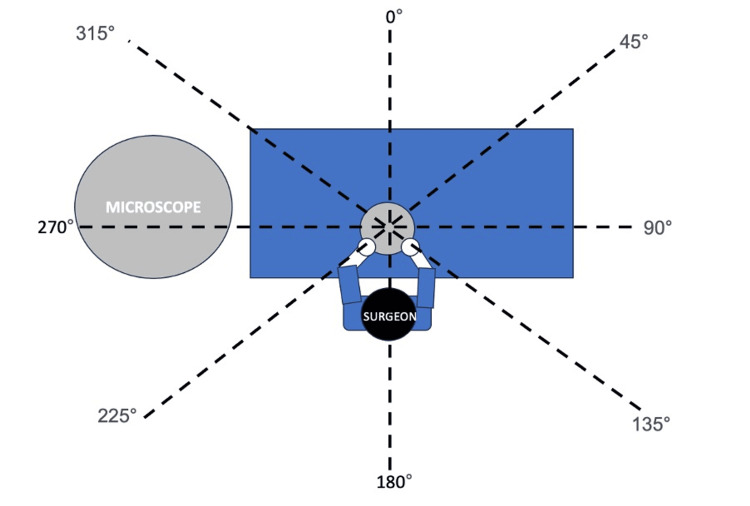
Experimental set-up. Grey circle ‘A’ refers to the cadaveric head. The cross-section aligns with the ear canal. The microscope was positioned in conditions B(microscope alone) and C (microscope fitted with Otodome), and absent in Condition A.

**Figure 2:**
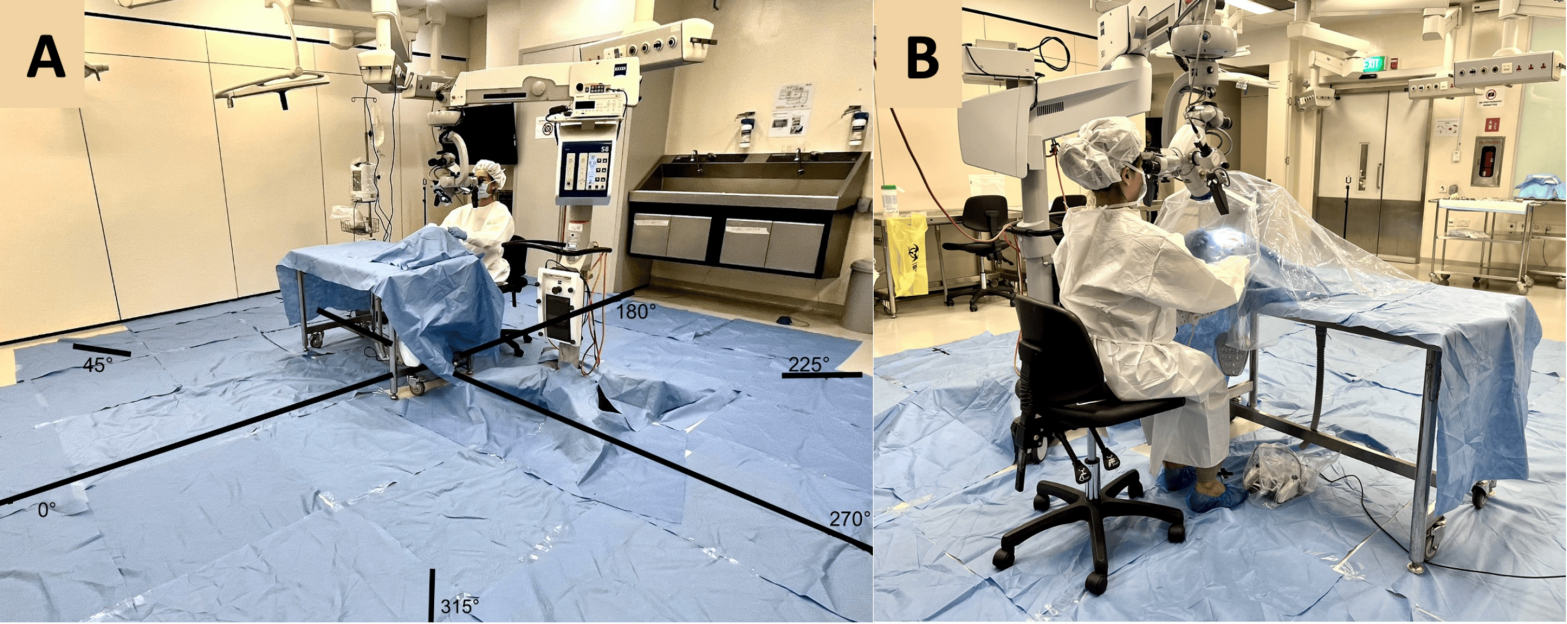
Experimental set-up in Conditions B and C A: Experimental set-up under condition B, using the microscope alone, B: Experimental set-up under condition C, i.e. where the microscope is fitted with the Otodome.

Fresh frozen cadaveric head specimens were prepared and mounted for dissection using the set-up as shown in Figure 1. The microscope was placed at the head of the table. 8 different markings were made circumferentially around the operative site at 0°,45°, 90°, 135°, 180°, 225°, 270°, 315° and 360° with the operative site being the centre of the axis and the surgeon seated at 180°. For this experiment, all 3 setups utilised the right ear of the cadaver. The same surgeon worked in all three scenarios and completed the cortical mastoidectomy in 12±1 minutes. Standard postauricular incisions were made, and the mastoid cortex was exposed. Mastoid drilling was performed with the Midas Rex Legend Drill (Medtronic, Minneapolis, USA) set at 60,000 revolutions per minute, with a 6 mm cutting drill bit. An integrated irrigation system was used, with Schülke optics fluorescent solution (Schülke & Mayr, Norderstedt, Germany)as irrigation fluid and irrigation speed set to 37 ml/minute. The same surgeon performed a cortical mastoidectomy under three conditions: A) without a microscope, with the surgeon wearing a face shield, B) with a microscope only (Figure 2A) and C) microscope with the attached Otodome (Figure 2B) (Figure 2A represents setup B). The Otodome is designed to be quickly and easily fitted to the microscope via a snap magnetic attachment, draping over the entire surgical field while allowing freedom of microscope movement. Further details of the design of the drape are beyond the scope of this manuscript.

After each cortical mastoidectomy, the extent of contamination of the surgeon and the surroundings was elucidated with ultraviolet LED (wavelength 365nm). The furthest distance that droplets travelled from the operative site (point A) was measured at the 8 different circumferential points as described above (Figure 1). 

Statistical analysis using SPSS version 27 was performed. Maximum dispersion of droplets under the various conditions was presented as median and interquartile range (IQR) and compared using the Mann-Whitney U test. Nonparametric tests were used due to the non-normal distribution of values.

Additionally, the density of contamination at 30 cm and 60 cm from point A was determined, and classified as mild, moderate or severe, where mild was defined as few scattered droplets (Figure 7 (appendix)). Moderate was defined as multiple droplets that were not coalescent (Figure 8 (appendix)). Severe was defined as coalescent droplets detected with significant amounts of bone dust present (Figure 9 (appendix)). A single blinded observer recorded these. Representative examples of this are included in the appendix.

The presence of contamination of the surgeon’s face/ mask area, head, chest, lap, right arm and left arm was recorded in each drilling condition.

## Results

Distance of droplet dispersion

**Figure 3:**
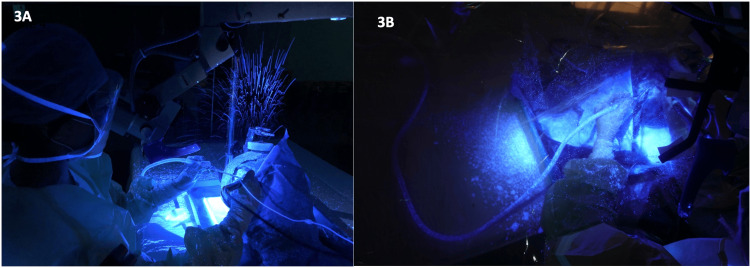
Images under ultraviolet LED light (wavelength 365nm) showing splash generated by droplet generation. A: Extensive splash created by mastoid drilling; B: The Otodome catching splash and droplets.

**Figure 4:**
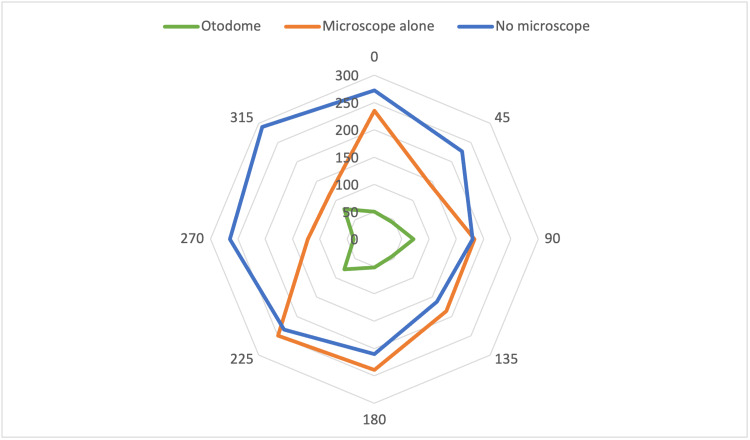
Maximum trajectory of droplets from operative site ‘A’. Blue refers to Condition A (no microscope). Orange refers to condition B (microscope alone). Green refers to condition C (Microscope was fitted with the Otodome).

In condition A, in which the surgeon performed the cortical mastoidectomy without the presence of the microscope, there was an extensive amount of droplet generation (Figure 3A). It showed the greatest area of contamination, with the greatest trajectory of droplets anterior and 45° to the left of the surgeon, at 272 to 290 cm (Figure 4, Table 1).

**Table 1:** Maximal trajectory from which droplets were detected from Point A under the 3 test conditions, namely A, without a microscope, B, with the microscope alone and C, with a microscope fitted with the novel barrier drape, Otodome. These were measured at 8 different points circumferentially. The surgeon was seated at 180 degrees.

Degrees	Condition A(cm)	Condition B(cm)	Condition C(cm)
0	272	235	50
45	227	143	45
90	180	183	72
135	162	186	45
180	210	239	52
225	234	250	78
270	265	122	38
315	290	116	78
Median(IQR)	230.5(187.5-270.3)	184.5(127.3-238.0)	51(41-76.5)

The presence of the microscope (without the barrier drape), i.e., Condition B, significantly attenuated the trajectory of the droplets to the left of the surgeon (Figure 4) due to its location at the head of the bed (Figure 1). However, there continued to be contamination from 0-225° with a maximum trajectory of 239 cm at 180°.

Finally, in condition C, where the microscope was fitted with the Otodome, the barrier drape was effective in catching splashes and droplets generated (Figure 3B). The spread of droplets was significantly attenuated in all directions, with the maximum trajectory being only 78cm (Figure 4, Table 1).

The distance of maximal dispersion was 230.5cm (IQR=187.5-270.3cm), 184.5cm (IQR=127.3-238.0cm) and 51cm (IQR=41-76.5cm) for conditions A, B and C, respectively. The distance of maximal dispersion was significantly reduced in Condition C (with the Otodome) compared to Condition B (U=0, p<0.001) and Condition A (U=0, p<0.001).

Density of droplet dispersion

**Figure 5:**
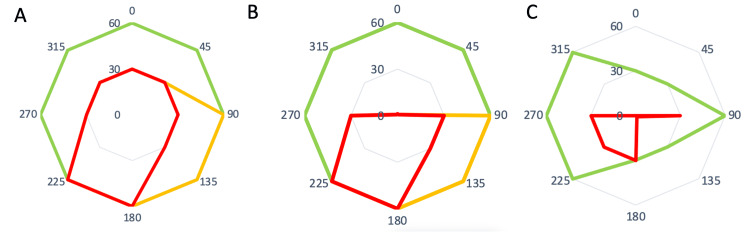
Degree of contamination at 30 cm and 60 cm from point A in all 3 conditions. Condition A (without microscope), Condition B (with microscope alone), Condition C(Microscope fitted with Otodome) Green refers to mild contamination. Orange refers to moderate contamination. Red refers to severe contamination.

In Condition A, the greatest degree of heavy contamination was noted. Heavy contamination was present in the entire field, 30 cm from the operative site. At 60 cm, heavy contamination was noted at 180° and 225°, while moderate contamination was noted at 90° and 135° (Figure 5A). 

In Condition B, there was only mild contamination of both quadrants anterior to the surgeon, i.e., 0-90° and 270-0° (Figure 5B). In condition C, heavy contamination was only noted in 4 of 8 points within the 30 cm diameter (Figure 5C), and only mild contamination was noted at 4 of 8 points in the 60 cm radius.

Droplet contamination of the surgeon

**Figure 6:**
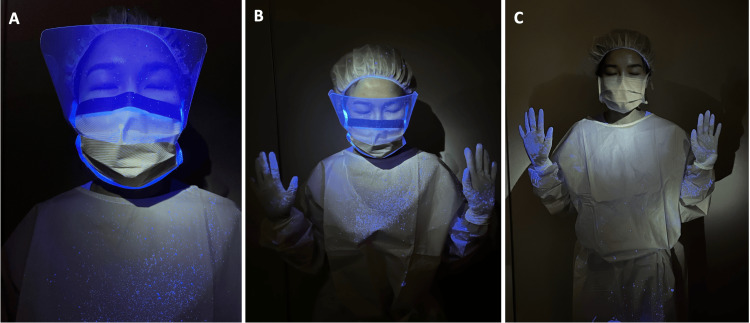
Surgeon contamination 6A, 6B are figures showing significant droplet splatter onto the surgeon after mastoidectomy was performed under Condition A ( without microscope), including critical areas such as on their head, mask and face ( despite use of faceshield). 6C: With the use of the Otodome, the degree of droplet splatter onto the surgeon was significantly reduced in the chest area with no noticeable contamination of the head, face and mask areas.

**Figure 7:**
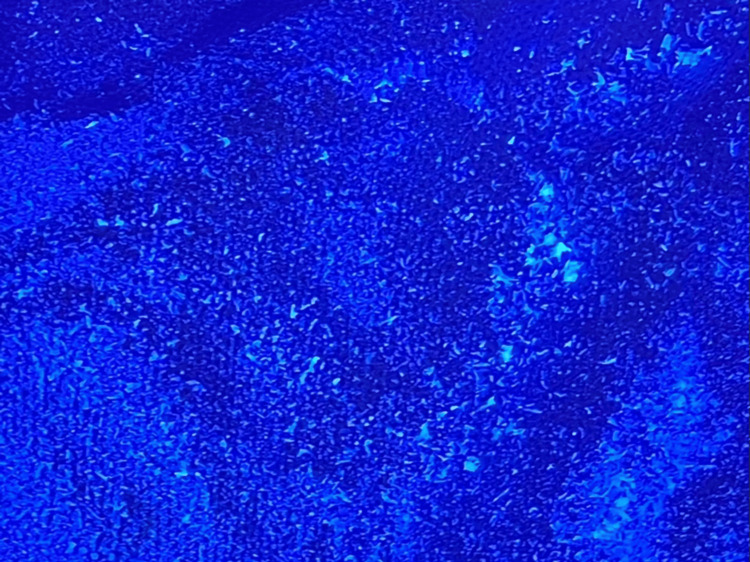
Example of mild contamination, i.e. few droplets are present without coalescence

**Figure 8:**
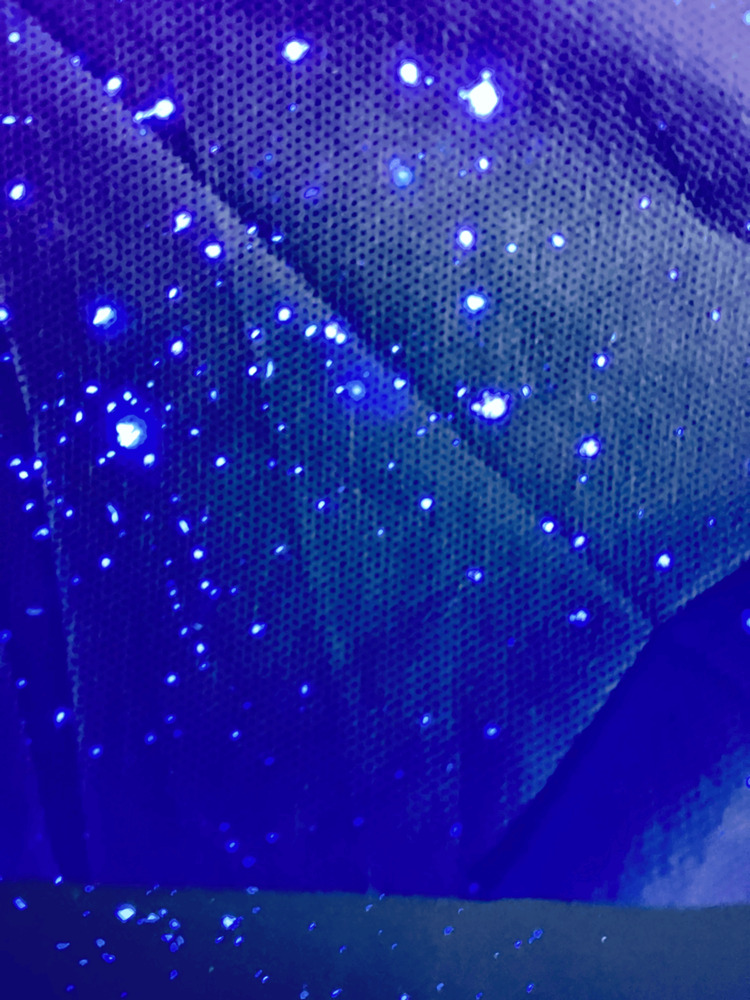
Example of moderate contamination i.e. multiple droplets present without coalescence.

**Figure 9:**
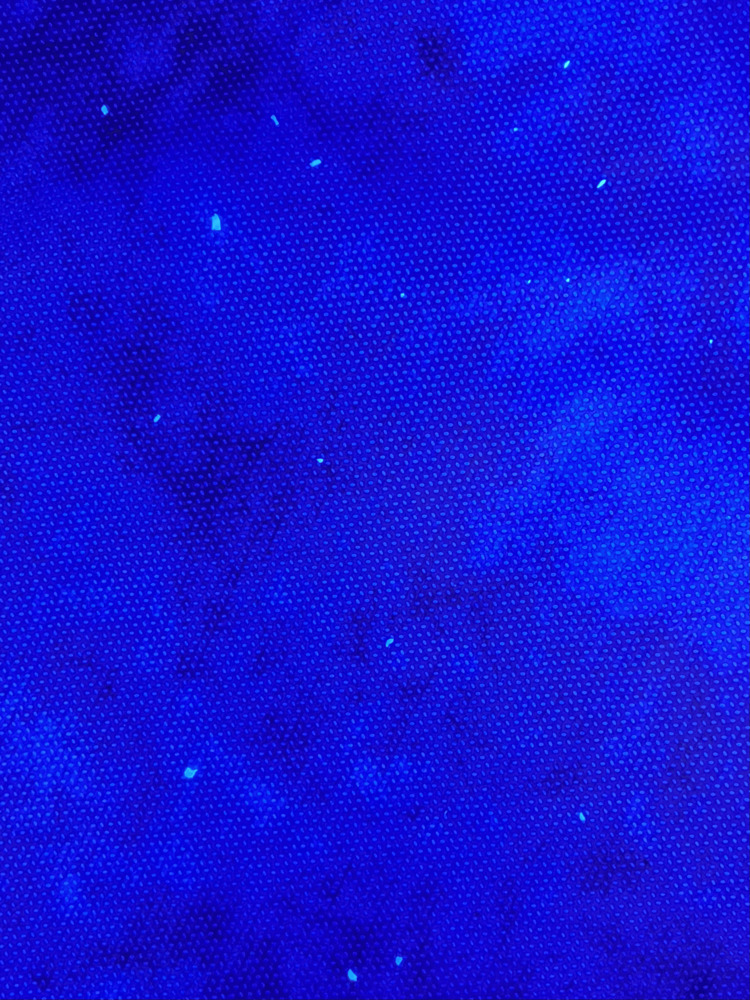
Example of severe contamination, where coalescent droplets were detected with significant amounts of bone dust present

In condition A, there was moderate to heavy contamination of the surgeon, with moderate contamination of critical areas, including the surgeon’s scrub cap and face (Figure 6, Table 2). Notably, despite the use of a face shield, there was still apparent contamination of the surgeon’s face by the droplets (Figures 6A, 6B).

The presence of the microscope in Condition B reduced the amount of droplets on the surgeon’s face, with only mild contamination. However, there continued to be heavy contamination of the surgeon’s chest, lap and both arms (Table 2).

With the use of the Otodome (Condition C), there was also no visible contamination of the head, face or mask regions of the surgeon, with mild contamination of the surgeon’s chest and moderate contamination of the surgeon's lap (Figure 6C).

**Table 2:** Degree of contamination on different subsites of the surgeon in the three conditions. Condition A, where no microscope was used for the cortical mastoidectomy. Condition B, where the microscope was used alone and Condition C, where the microscope was fitted with the novel barrier drape, Otodome.

Location	Condition A	Condition B	Condition C
Head	Moderate	Mild	None
Face/Mask	Moderate	Mild	None
Chest	Heavy	Heavy	Mild
Right arm	Heavy	Heavy	Heavy
Left arm	Heavy	Heavy	Heavy
Lap	Heavy	Heavy	Moderate

## Discussion

This study adds to existing recent literature, which shows that extensive droplet and/or aerosol dispersion occurs during mastoid surgery [2,3,6,7]. Cottrell et al. showed that while drilling under a microscope, particles were dispersed up to 68 inches (172.2cm) away from the operative field [6]. This study similarly found droplets dispersed a significant 290cm from the operative field. The difference in the actual distance measured is possibly related to the differences in the drill speed used, as this study utilised a 60000 RPM drill while Cottrell et al. utilised a 70000 RPM drill. Other studies used different methods of quantifying particle and aerosol dispersion. Chiari et al. used an optical particle sizer, which detects particles less than 10 μm, and found that 61500 particles per litre were generated 30cm from the operative site [2]. Freiser et al. similarly utilised a cascade impact to quantify aerosolisation during mastoidectomy [7]. Their study found that aerosols under 14.1 μm were produced during mastoidectomy. 

Our experimental set-up was similar to that used by a study by Markey et al., though notably the speed of the drill and the rate of irrigation were not mentioned in that study [8]. In our study, we had the additional advantage of including the density of droplets generated at 2 separate distances from the operative set-up. The 30 cm radius reflects the spread of droplets and bone dust in the immediate surroundings of the patient, while the 60 cm radius addresses potential contamination of surgical assistants or the scrub nurse. As seen in Figure 5, there was at least moderate contamination of a 60 cm radius from 90-225° in condition A, where no microscope was used. In contrast, the use of the barrier drape confined most of the heavy contamination to be within a limited area (30 cm radius), as this was the area under the barrier drape. Sharma et al. determined that in their study, there was gross contamination up to 6 feet from the surgical site, with individuals such as surgical assistants within 1 to 3 feet of the drill being at an increased risk of exposure [9]. Typically, in our usual operating set-up, the scrub nurse would be on the opposite side of the patient, facing the surgeon, corresponding to position 315-0°. The significant reduction in the contamination at this location highlights that the Otodome is not only helpful in preventing the contamination of the primary surgeon but also significantly reduces the risks of contamination to the rest of the surgical team.

The most commonly proposed solution to mitigate the risk of infection from droplet and aerosol dispersion during mastoidectomy is the use of improvised barrier drapes to contain the surgical field. Das et al. proposed a two-draped closed pocket technique using a modified version of an existing opaque drape [4]. Chen et al. demonstrated the use of an existing 1060 Steri-Drape cut to fit the microscope lens [3]. Cottrell et al. similarly described the use of readily available equipment to create a barrier system, which was utilised for urgent neurotology cases during a pandemic [6]. The above solutions have proved invaluable as quick solutions were being sought when the COVID-19 pandemic first highlighted various loopholes in infection control practices. However, the use of improvised drapes is less ideal in the longer term as they require modification and are more time-consuming to apply. Excess drape material may also interfere with the dexterity of the surgeon during surgery and increase operative times. 

To circumvent these issues, this study utilised a custom-made prototype drape that can be attached securely to the operative microscope. The design of the drape is not elaborated in this study, which is aimed to be a proof-of-concept study demonstrating the utility of a novel barrier drape in mitigating the degree of splash and droplet dispersion during mastoidectomy. There was a significant reduction in the droplet distribution with the use of the Otodome as compared to the two other conditions, with the microscope alone and without a microscope. Importantly, the use of the Otodome prevented any droplet generation onto critical areas, such as the surgeon's head, face and mask areas. Lawrence et al. also described the use of a “drape “tent, where the tent was attached to the microscope and the operating table and extended to cover the patient down to the level of the lower abdomen. In this study, no macroscopic droplet spread or particulate matter outside the tent was visualised; however, no objective measurements were taken [10]. There was only 1 other study by Chari et al., which utilised custom prototypes, named OtoTent 1 and OtoTent 2 [3], which is now commercially available as Otoshield (Grace Medical, Memphis, USA). Although there are major differences in the design of the prototype, these drapes appeared to be easy to use and effective. The predominant difference between Otoshield and our novel barrier drape design is that Otoshield is secured circumferentially around the surgical field after being attached to the microscope - this limits the extent of movement of the microscope, but creates a greater degree of seal around it. Additionally, our drapes are easily set up by means of magnets, which attach easily to the microscope.

A limitation of the Otodome is that it does not create an airtight seal around the operative site. As a result, it does not absolutely eliminate the risk of droplet and aerosol dispersion during surgery. However, the design of a drape that would create an airtight seal around the operative site would be likely to hinder with passage of instruments during surgery and also be more cumbersome to use, as any movement allowed with the microscope will be limited. As such, a compromise between mitigating risks of transmission of infectious agents and creating a user-friendly drape has to be made. 

Although this was not the primary outcome measure of this study, the surgeon using the Otodome also noted that the drape was quick to set up and did not affect visualisation or hinder dexterity during surgery. Additionally, in comparison, using a face shield to prevent splash on the surgeon would often make visualisation suboptimal due to its reflective surface and accumulation of splash and bone dust on the face shield, particularly when using a microscope.

Future directions include further studies on the “usability” of the drapes to assess the ease of set-up and usage by different surgeons. This is an important consideration to assess our product to determine if it is sustainable for long-term use. Our product may also be adapted in the future for other subspecialties that also utilise drilling procedures, including orthopaedics, plastics and reconstructive surgery and oromaxillofacial surgery.

## Conclusions

This study demonstrates that mastoidectomy produces an extensive amount of droplet dispersion onto both the surgical team and their surroundings. This can be mitigated by the use of the Otodome. Further development of the prototype, commercialisation and usability studies will be undertaken.

## References

[1] Chen JX, Workman AD, Chari DA (2020). Demonstration and mitigation of aerosol and particle dispersion during mastoidectomy relevant to the COVID-19 era. Otol Neurotol.

[2] Lawrence RJ, O'Donoghue G, Kitterick P (2020). Recommended personal protective equipment for cochlear implant and other mastoid surgery during the COVID-19 era. Laryngoscope.

[3] Chari DA, Workman AD, Chen JX (2021). Aerosol dispersion during mastoidectomy and custom mitigation strategies for otologic surgery in the COVID-19 era. Otolaryngol Head Neck Surg.

[4] Das A, Mitra S, Kumar S, Sengupta A (2020). Two-drape closed pocket technique: Minimizing aerosolization in mastoid exploration during COVID-19 pandemic. Eur Arch Otorhinolaryngol.

[5] Lawrence RJ, O'Donoghue GM, Kitterick P (2020). Use of a novel drape 'tent' as an infection prevention control measure for mastoid surgery. J Laryngol Otol.

[6] Cottrell J, Lui J, Le T (2020). An operative barrier system for skull base and mastoid surgery: Creating a safe operative theatre in the era of COVID-19. J Otolaryngol Head Neck Surg.

[7] Freiser ME, Dharmarajan H, Boorgu DSSK, Edward S, Timothy C, Jabbour N, David HC (2021). Droplet and aerosol generation with mastoidectomy during the COVID-19 pandemic: Assessment of baseline risk and mitigation measures with a high-performance cascade impactor. Otol Neurotol.

[8] Markey AL, Leong SC, Vaughan C (2021). Droplet and bone dust contamination from high-speed drilling during mastoidectomy. Clin Otolaryngol.

[9] Sharma D, Rubel KE, Ye MJ (2020). Cadaveric simulation of Otologic procedures: An analysis of droplet splatter patterns during the COVID-19 pandemic. Otolaryngol Head Neck Surg.

[10] Lawrence RJ, O'Donoghue GM, Kitterick P, Hartley DE (2020). Use of a novel drape 'tent' as an infection prevention control measure for mastoid surgery. J Laryngol Otol.

